# Macular Retinal Microvasculature of Hyperopia, Emmetropia, and Myopia in Children

**DOI:** 10.3389/fmed.2022.900486

**Published:** 2022-05-20

**Authors:** Liang Lv, Mu Li, Xuejiao Chang, Mengxia Zhu, Ying Liu, Ping Wang, Yan Xiang

**Affiliations:** ^1^Department of Ophthalmology, Tongji Hospital, Tongji Medical College, Huazhong University of Science and Technology, Wuhan, China; ^2^Department of Ophthalmology, Hankou Aier Eye Hospital, Wuhan, China; ^3^Department of Ophthalmology, Wuhan Union Hospital, Tongji Medical College, Huazhong University of Science and Technology, Wuhan, China

**Keywords:** myopia, macular retinal microvasculature, optical coherence tomography angiography, deep capillary plexus, superficial vascular plexus

## Abstract

**Purpose:**

To compare macular retinal microcirculation in myopia, emmetropia, and hyperopia groups and investigate the relationship between macular retinal microcirculation and axial length (AL) in children.

**Methods:**

Forty myopic, 29 emmetropic, and 34 hyperopic eyes were included. All the recruited eyes underwent optical coherence tomography angiography (OCTA) examinations. After adjusting the image size by the Littmann method and Bennett formula, the vessel density (VD) of the deep capillary plexus (DCP) and superficial vascular plexus (SVP) were assessed.

**Results:**

The VD of the DCP was significantly lower in the myopia group than in the hyperopia group, whereas no significant differences in the VD of the SVP were observed among the myopia, emmetropia, and hyperopia groups. The VD of the DCP was significantly associated with AL, spherical equivalent (SE), and foveal retinal thickness (FRT), whereas the VD of the SVP was only significantly associated with FRT but not with AL or SE.

**Conclusions:**

The myopic VD of the DCP was significantly lower than the hyperopic one, and the VD of the DCP was significantly associated with AL, indicating that myopia has a lower VD of the DCP, and AL could have a negative effect on the VD of the DCP. Thus, early myopic axial stretching might decrease retinal blood perfusion of the DCP in children.

## Introduction

The global incidence of myopia was reported to be 28.3% in 2010 and is estimated to reach 52% by 2050 (1). There is an epidemic of myopia in East and Southeast Asia, where the prevalence of myopia and high myopia in young adults is 80–90% and 10–20%, respectively (2). Myopia is characterized by excessive axial elongation, which can stretch the retina, resulting in retinal thinning, vitreoretinopathy, and even blindness (3–5). The increasing prevalence of myopia emphasizes the importance of prevention and control of its development.

The retina has the highest metabolic demand among body tissues (6). Normal visual function is highly dependent on effective retinal blood perfusion, and axial elongation and stretching may affect retinal blood perfusion in myopic eyes (7). Previous studies have used various methods to investigate the retinal vascular changes in myopia. Shimada et al. (8) found a reduction in retinal blood flow, and Benavente-Perez et al. (9) reported a reduction in pulse amplitude in the central retinal artery by Doppler instrument in the high myopia group. Azemin et al. found a decrease in large retinal vessel density (VD) in myopic eyes using the NAVIS Lite Image Filing System (10), and Lim et al. found decreases in retinal VD in myopic eyes using retinal fundus photographs (11).

With the development of optical coherence tomography angiography (OCTA), we can now observe the retinal microcirculation *in vivo* and in separate sublayers (12, 13). In addition, quantitative OCTA measurements show good reliability and repeatability (14). Using spectral-domain OCTA, numerous studies have indicated alterations in the retinal microcirculation in myopic eyes. Previous studies have suggested that both the VD of the superficial vascular plexus (SVP) and deep capillary plexus (DCP) decrease in the macular area of high myopic eyes (15, 16). He et al. found that the parafoveal VD of the DCP decreased significantly, whereas the parafoveal VD of the SVP remained unchanged in high myopia (17). Moreover, Milani et al. indicated a decrease in the VD of the SVP and an increase in the outer retinal flow in myopic eyes (18). However, these studies were mainly conducted in adults, and little is known about the myopic changes in retinal microcirculation among childhood, which is the main stage of myopia onset and development (19–21). The investigation of myopia-related retinal vessel changes early in childhood (the onset of myopia) might provide more information about myopia than later in the adult stage. Although Gołębiewska et al. (19) studied the myopic retinal VD in children, they only measured superficial retinal VD, but no deep retinal VD. Previous studies have indicated that the deep retinal VD might be more sensitive to the myopic changes than the superficial retinal VD (15, 17, 22, 23). Thus, superficial retinal VD measurements alone is insufficient for myopic retinal microvasculature studies. Moreover, they (19) only included emmetropic and myopic eyes but no hyperopic eyes in their study, leading to the limited refractive ranges of their study subjects.

Accordingly, in this study, we aimed to compare macular retinal microcirculation in both the DCP and SVP sublayers among a wider range of refractive groups (hyperopia, emmetropia, and myopia), and to investigate the relationship between macular retinal microcirculation and axial length (AL) in children.

## Materials and Methods

The study was approved by the Ethics Committee of Tongji Hospital, Tongji Medical College, Huazhong University of Science and Technology (TJ-IRB20190417), and adhered to the tenets of the Declaration of Helsinki. The guardians of the enrolled children signed written informed consent before participation.

### Subjects

This study recruited 62 children from June 2019 to June 2020 at the optometry clinic of Tongji Hospital. Both eyes of each participant were included for ophthalmologic examinations: best-corrected visual acuity (BCVA) assessment (Snellen chart), cycloplegic optometry (1% cyclopentolate was instilled 30 and 25 min before optometry/autorefraction), comprehensive optometry, intraocular pressure measurement (non-contact tonometer, RT-2100; Nidek Co., Ltd., Gamagori, Aichi, Japan), AL measurement (IOL-Master 500; Carl Zeiss Meditec, Dublin, CA, United States), fundus photography (AFC-210, Nidek Co., Ltd., Gamagori, Aichi, Japan), and slit-lamp examination. Subjects with amblyopia or squint (24), ocular diseases other than refractive error, a history of ocular trauma, a history of ocular laser or surgery, BCVA < 6/6, astigmatism ≥ 1.00 diopters (D), wearing orthokeratology lenses, or systemic disease were excluded from participation.

Among the recruited 124 eyes from 62 children, 21 were excluded due to low OCTA image quality. Low OCTA image quality was evaluated by (1) quality index < 40, (2) definite motion artifacts, (3) ambiguous segmentation of retinal layers, (4) poor centering, or (5) signal loss during blinking (25, 26). According to cycloplegic optometry results, the remaining eyes were divided into hyperopia, emmetropia, and myopia groups based on the following criteria: spherical equivalent (SE) > 0.5 D as hyperopia, –0.5 D ≤ SE ≤ 0.5 D as emmetropia, and SE < –0.5 D as myopia (19). Thus, we recruited 40 myopic eyes from 29 children, 29 emmetropic eyes from 22 children, and 34 hyperopic eyes from 20 children in this study.

### Image Acquisition

The participants were scanned using a Spectralis^®^ OCT Angiography Module (Heidelberg Engineering, Heidelberg, Germany) with a 3 mm × 3 mm scan area centered on the fovea. The Spectralis^®^ OCT Angiography Module provides high-resolution OCTA images with a lateral resolution of 5.7 μm/pixel and an axial resolution of 3.9 μm/pixel. The laser wavelength was 870 nm and the scan rate was 40 kHz. Moreover, it can average the images and reduce the noise with the automated real-time mode and provide high-quality angiogram in case of poor fixation or blinking with the active eye-tracking system. The manufacturer’s proprietary 2D projection artifact removal algorithm was used to allow for quantitative retinal vascular assessments. After image acquisition, the built-in software automatically calculated macular retinal thickness and separated the retinal blood vessels into four sublayers: nerve fiber vascular plexus, SVP, intermediate capillary plexus, and DCP. This segmentation of the four sublayers was based on the proposal raised by Campell et al. (27). In this study, the SVP and DCP were selected for further analysis.

### Image Analysis

**FIGURE 1:**
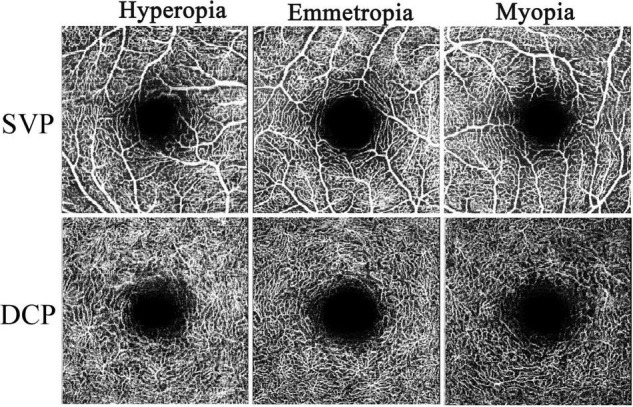
Representative OCTA images of SVP and DCP in hyperopia, emmetropia, and myopia groups.

**FIGURE 2:**
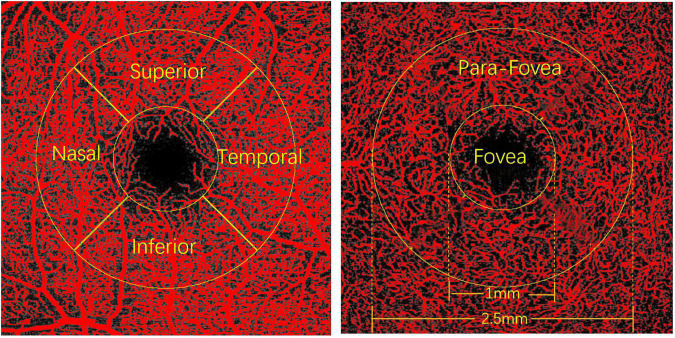
The diagram of the parafoveal area.

The obtained images of the SVP and DCP were exported from the Spectralis^®^ OCT Angiography Module in TIF format and were further analyzed using FIJI software (an expanded version of ImageJ version 1.51a^1^) (Figure 1). As the image magnification and VD would be impacted by AL, we adjusted the image size according to the individual ALs using the Littmann method and Bennett formula to all the study participants (28–30). The image from Spectralis^®^ OCT Angiography Module was corrected by the correction factor 3.393 × 0.01306 × (AL-1.82) (linear magnification) or 3.393^2^ × 0.01306^2^ × (AL-1.82)^2^(area magnification) (31, 32). After magnification correction, OCTA images were binarized for further VD of the SVP and DCP assessments. VD was defined as the percentage of the area occupied by the retinal vessels. The binarization was performed with intensity thresholding using Otsu’s thresholding method as integrated into the FIJI software (33). Based on previous studies (34, 35), two concentric circles (diameter of 1 mm and diameter of 2.5 mm) centered on the fovea were selected. The area between the inner and outer circles is defined as the parafoveal area. The parafoveal area was further divided into four parts: superior, inferior, nasal, and temporal quadrants (Figure 2). Partition of the retinal area was performed using the ROI Manager feature in the FIJI software. The image was converted into an 8-bit image to allow the application of the Otsu auto local threshold tool. The retinal vessel of the binarized image was highlighted in white using the threshold tool. Image partitions are pre-set and recorded in the ROI Manager, including the parafovea and its four quadrants. After selecting the corresponding partition in ROI Manager, VD was measured by selecting the “limit to Threshold” option (Supplementary Figure 1). In terms of foveal avascular zone (FAZ) measurement, according to the study of Ishii et al. (36), the Kanno-Saitama Macro program is an automated analysis program of ImageJ macro, which can extract the FAZ automatically. Subsequently, the FAZ area was calculated. All data collection, measurements, and analyses were blinded to the study subject information.

### Statistical Analysis

All analyses were performed by SPSS software 21.0 (IBM Corp., Armonk, NY, United States). Data were shown as mean ± SD where applicable. Analysis of variance was used for the intergroup comparisons of age. Chi-square statistic was used for the intergroup comparisons of sex. Generalized estimating equations (GEEs), which take the correlation of measurements of two eyes of one subject into account, were used for the intergroup comparisons of AL, SE, foveal retinal thickness (FRT), BCVA, FAZ, and parafoveal VD of DCP and SVP. The associations between parafoveal VD and AL, SE, FRT, and age were also estimated by GEEs.

## Results

There were no significant differences in age, sex, or BCVA among the hyperopia, emmetropia, and myopia groups (all *p* > 0.05). The average AL value of all the study subjects was 23.51 ± 1.30 mm (range, 21.15–26.19 mm), and the average SE value of all the study subjects was 0.25 ± 3.11 D (range, –6.75 D to +6.0 D). The three refractive subgroups showed significant differences in terms of AL, SE, and FRT, with the hyperopia group having the shortest AL, highest SE, and thickest FRT, whereas the myopia group had the longest AL, lowest SE, and thinnest FRT (all *p* < 0.05) (Table 1).

**TABLE 1:** Study subject characteristics.

	Hyperopia	Emmetropia	Myopia	*P*1	*P*2	*P*3
Age (years)	10.1 ± 2.5	11.0 ± 2.2	10.7 ± 1.8	0.153	0.341	0.554
Sex (male/female)	10/10	10/12	11/18	0.768	0.401	0.589
Axial length (mm)	22.14 ± 0.79	23.66 ± 0.74	24.57 ± 0.85	<0.001*	<0.001*	<0.001*
Spherical equivalent (D)	+3.99 ± 1.49	+0.03 ± 0.13	–2.76 ± 1.62	<0.001*	<0.001*	<0.001*
Foveal retinal thickness (μm)	280.6 ± 15.2	271.0 ± 16.5	263.3 ± 12.4	0.076	<0.001*	0.086
Best corrected visual acuity	1.04 ± 0.08	1.04 ± 0.08	1.02 ± 0.07	0.750	0.599	0.368

P1: Comparison between hyperopia and emmetropia; P2: Comparison between hyperopia and myopia; P3: Comparison between emmetropia and myopia. Analysis of variance for the intergroup comparison of age; Chi-square statistic for the intergroup comparison of sex; GEEs for the intergroup comparison of axial length, spherical equivalent, foveal retinal thickness, and best corrected visual acuity. *Significance of difference.

### The Comparisons of Parafoveal Retinal Vessel Density and Foveal Avascular Zone Among Hyperopia, Emmetropia, and Myopia Groups

For DCP, the average and quadrantal (superior, inferior, nasal, and temporal) values of the parafoveal VD showed significant differences between the hyperopia and myopia groups (all *p* < 0.05). Other than the inferior parafoveal VD comparison between hyperopia and emmetropia, the temporal parafoveal VD comparisons between hyperopia/myopia and emmetropia, there were no significant parafoveal VD (both average and quadrantal values) differences between the hyperopia/myopia and emmetropia groups (all *p* > 0.05). For SVP, no significant differences in parafoveal VD were found among the hyperopia, emmetropia, and myopia groups (all *p* > 0.05) (Table 2).

**TABLE 2:** The comparisons of parafoveal retinal vessel density among hyperopia, emmetropia, and myopia groups.

Parafoveal vessel density (VD) of deep capillary plexus (DCP) (%)						
	**Hyperopia**	**Emmetropia**	**Myopia**	***P*1**	***P*2**	***P*3**
Superior	39.0 ± 4.9	37.3 ± 6.2	34.6 ± 5.4	0.298	0.001*	0.084
Inferior	36.4 ± 6.0	32.2 ± 5.4	31.0 ± 5.8	0.013*	0.001*	0.433
Nasal	35.7 ± 5.7	34.5 ± 7.7	32.2 ± 5.0	0.550	0.009*	0.262
Temporal	35.5 ± 5.9	32.4 ± 5.0	29.7 ± 5.1	0.033*	<0.001*	0.034*
Average	36.5 ± 4.5	34.0 ± 5.1	31.8 ± 3.6	0.079	<0.001*	0.086

**Parafoveal vessel density (VD) of superficial vascular plexus (SVP) (%)**						

	**Hyperopia**	**Emmetropia**	**Myopia**	***P*1**	***P*2**	***P*3**

Superior	44.4 ± 6.1	43.6 ± 7.0	43.9 ± 5.9	0.644	0.768	0.837
Inferior	45.2 ± 5.2	43.9 ± 6.7	43.1 ± 6.1	0.462	0.170	0.670
Nasal	45.2 ± 7.4	42.7 ± 8.7	44.5 ± 7.2	0.269	0.676	0.415
Temporal	42.7 ± 5.1	42.2 ± 6.7	40.6 ± 6.0	0.776	0.129	0.368
Average	44.3 ± 4.8	43.0 ± 6.6	43.0 ± 5.2	0.138	0.293	0.682

P1: Comparison between hyperopia and emmetropia; P2: Comparison between hyperopia and myopia; P3: Comparison between emmetropia and myopia. *Significance of difference. GEEs for the intergroup comparisons. Age and sex have been adjusted.

*Significance of difference.

Both superficial and deep FAZ showed no significant differences among the hyperopia, emmetropia, and myopia groups (all *p* > 0.05) (Table 3).

**TABLE 3:** The comparisons of superficial and deep foveal avascular zone among hyperopia, emmetropia, and myopia groups.

	Hyperopia	Emmetropia	Myopia	*P*1	*P*2	*P*3
Superficial FAZ (mm^2^)	0.41 ± 0.06	0.43 ± 0.05	0.40 ± 0.04	0.385	0.403	0.071
Deep FAZ (mm^2^)	0.41 ± 0.04	0.42 ± 0.04	0.40 ± 0.04	0.412	0.380	0.094

*P1: Comparison between hyperopia and emmetropia; P2: Comparison between hyperopia and myopia; P3: Comparison between emmetropia and myopia. GEEs for the intergroup comparisons. Age and sex have been adjusted. FAZ, foveal avascular zone.*

### Univariate Regression Between Parafoveal Retinal Vessel Density and Axial Length, Spherical Equivalent, Foveal Retinal Thickness, Age

In the DCP sublayer, all VD values were significantly associated with AL and SE (all *p* < 0.05). The superior, inferior, nasal, and average VDs were significantly associated with FRT (all *p* < 0.05). Although quadrantal VD values were not significantly associated with age (all *p* > 0.05), the average VD value was found to be significantly associated with age (*p* = 0.049).

In the SVP sublayer, no significant associations between VD and AL, and SE were found (all *p* > 0.05). Similar to the results of the DCP, the superior, inferior, nasal, and average VDs were significantly associated with FRT (all *p* < 0.05). Moreover, only superior VD was significantly associated with age (*p* = 0.017) (Table 4).

**TABLE 4:** Univariate regression between parafoveal retinal vessel density and axial length, spherical equivalent, foveal retinal thickness, age.

	AL (mm)		SE (D)		FRT (μm)		Age (years)	
VD of DCP (%)	β	*p*	β	*p*	β	*p*	β	*p*
Superior	–8.24	<0.001*	17.05	0.002*	75.50	0.032*	–7.39	0.062
Inferior	–5.73	0.011*	19.65	<0.001*	72.30	0.033*	–6.25	0.126
Nasal	–5.05	0.031*	13.99	0.013*	100.01	0.010*	–5.01	0.237
Temporal	–8.14	<0.001*	21.12	<0.001*	60.12	0.069	–8.18	0.056
Average	–10.85	<0.001*	29.00	<0.001*	129.81	0.004*	–10.51	0.049*

	**AL (mm)**		**SE (D)**		**FRT(μm)**		**Age (years)**	
**VD of SVP (%)**	β	***P***	β	***P***	β	***P***	β	***P***

Superior	0.70	0.772	0.20	0.966	78.08	0.007*	–9.60	0.017*
Inferior	–2.87	0.194	6.00	0.209	81.12	0.023*	–8.89	0.054
Nasal	–1.21	0.549	1.98	0.607	63.86	0.027*	–6.05	0.123
Temporal	–0.84	0.722	2.57	0.649	56.34	0.129	–8.31	0.130
Average	–1.51	0.584	3.66	0.483	104.09	0.011*	–11.24	0.052

*β/P value: Regression coefficient and p values of the independent variables were calculated by GEEs. The influence factors as age and/or sex have been adjusted. VD, vessel density; DCP, deep capillary plexus; SVP, superficial vascular plexus.*

*Significance of difference.

## Discussion

In this study, we found that the VD of the DCP and FRT were significantly lower in the myopia group than in the hyperopia group, whereas no significant VD of SVP differences was observed among the hyperopia, emmetropia, and myopia groups. VD of the DCP was significantly associated with AL, SE, and FRT, whereas VD of the SVP was only significantly associated with FRT, but was not with AL or SE. Both superficial and deep FAZ showed no significant differences among the hyperopia, emmetropia, and myopia groups.

It has been reported that AL could affect the optical magnification, change the actual scan size, and potentially interfere with vessel caliber measurement (37, 38). A longer AL makes the retinal VD artificially denser because of the larger fundus area scanned under smaller magnification (39). For the magnification error correction, the only use of AL was accurate and produced results very similar to the more complex calculations, which take keratometry, refractive error, and lens thickness corrections into consideration (40). Accordingly, we adjusted the image size based on their individual ALs using the Littmann method and Bennett formula (28–30).

A previous study by He et al. reported that the deep parafoveal VD decreased significantly in eyes with high myopia, whereas no changes in the superficial parafoveal VD were observed. Moreover, the deep parafoveal VD was found to be significantly associated with AL (17). Similar results were also found by Lin et al., indicating that only the VD of the DCP showed a significant reduction, whereas the VD of the SVP showed no significant changes in high myopic eyes (41). Their results were consistent with our study results, which reported that the VD of the DCP was significantly lower in the myopia group than in the hyperopia group, whereas no significant VD of SVP differences was observed among the hyperopia, emmetropia, and myopia groups. The DCP might be the most essential perfusion plexus in the retina (23, 42). There were evidences suggesting that, compared with SVP, DCP is more related and vulnerable to myopia (15, 17, 22, 23). The underlying reason could be that the mechanical stretch caused by the excessive axial elongation of myopic eyes is more susceptible to influencing and disrupting the vulnerable small-diameter retinal vessels in the DCP than the large-diameter retinal vessels in the SVP (8, 34, 43, 44). In addition, DCP is reported to be difficult to recover from injury, which could also explain the significant decrease in the VD of DCP in myopia (23, 45, 46). This difference between DCP and SVP was also supported by the study of Lin et al., reporting that VD loss in the DCP was significantly faster in the high myopia group than in the normal group, whereas the rate of VD loss in the SVP was equal in high myopia and normal groups (41). Moreover, a previous study reported that VD of DCP changed significantly with aging, whereas VD of SVP remained relatively unchanged (13), indicating that VD of DCP could be more vulnerable to external influence factors than VD of SVP.

In addition to decreased VD of the DCP in myopia, the VD of the DCP was found to be significantly associated with AL, indicating that myopia and AL have significant negative effects on the VD of the DCP. Excessive axial elongation is the original mechanism for the development of myopia and can lead to the stretching and thinning of the ocular tissues, particularly the choroid and retina (4). The VD of retinal vessels is established at or shortly after birth (47), whereas myopia mainly develops at the age of 8–14 years old, which is much later than the period of retinal vessel formation (19–21). Thus, with the development of myopia and AL, retinal VD decreases because of the mismatch in the fixed vascular formation at birth and the increasing eyeball size later in childhood, which might further result in metabolic challenges in the retina (5, 34). Although the exact mechanism of decreased retinal VD in myopic eyes has not been elucidated, several explanations have been reported: (1) Elongation of the myopic eyeball could lead to stretching and thinning of the retina. Stretching of the retina could exert mechanical force on retinal vessels, resulting in partial vascular compromise and reduced VD. As the metabolic substrate, blood, and nutrient supplier to the retina, the reduction in retinal VD might cause further loss of retinal cells in return (5). Thus, the decreased VD of retinal vessels in myopia might be passive and pathological, owing to stretching of the retina from the elongation of the eyeball. (2) The elongation of the eyeball could stretch the retina, making the retina thinner. Considering the supply-demand principle, a thinner retina might require fewer vessels for metabolic substrate, blood, and nutrient supply, leading to the loss and atrophy of retinal vessels (5, 48). Thus, the decreased VD of retinal vessels in myopia might be active and physiological, following changes in the retina. (3) Vascular endothelial growth factor (VEGF) is produced by retinal vascular endothelial cells and retinal pigment epithelium cells and may play an important role in the formation of vessels and the development of retinal vasculature (49, 50). Elongation of the eyeball stretches the retina, making the retina thinner with the development of myopia. Following retinal thinning, degeneration of retinal vascular endothelial cells and retinal pigment epithelium cells occurs, leading to the relevant decreases in VEGF production (39). Thus, the decreased VEGF level by longer AL could also be one reason for the reduction in retinal VD in myopia. (4) Excessive elongation of the eyeball could stretch and redistribute the retina, leading to an increase in the retinal area. For retinal cells and vessels, increasing the retinal area could result in decreases in the density of retinal cells and vessels. Considering that the decreases in retinal cells and vessels were synchronous and comparable, the blood supply of retinal vessels seems to be sufficient for retinal metabolism (5).

The subjects in this study were children, as the main stage of myopia onset and development (19–21). Moreover, from the perspective of refractive status, most of the myopic eyes in this study were mild to moderate, which are also the early stage of myopia. Thus, the observed decreases in myopic VD of the DCP among children might indicate that decreases in myopic VD of the DCP could occur as early as the onset stage of myopia. Previous studies have reported that the DCP is the main blood supply to the deep retina, including the inner plexiform, inner nuclear, and outer plexiform layers (16, 27, 34, 51), which are suggested to be important loci of the myopia-related dopamine signaling pathway (52). Accordingly, we speculated that the decrease in VD of the DCP occurring early in the onset of myopia might promote the development of myopia by affecting the blood supply of the deep retina, and the following dopamine signaling pathway. However, this speculation requires more research to verify.

This study had certain limitations. First, our sample size was relatively small. Second, our study was cross-sectional but not longitudinal. Thus, the causality between decreased retinal thickness and retinal VD remains unclear. Third, childhood is the onset and development stage of myopia (19–21), making the incidence of high myopia relatively low in children. Therefore, we did not perform a subgroup analysis of high myopia in this study.

In conclusion, the myopic VD of the DCP was significantly lower than the hyperopic VD of the DCP, and the VD of the DCP was significantly associated with AL, indicating that myopia has a lower VD of the DCP, and AL could have a negative effect on the VD of the DCP. Early myopic axial stretching might cause a decrease in retinal blood perfusion of the DCP in children, and the differences in retinal microcirculation of the DCP between hyperopic and myopic eyes might be one potential reason for the development of myopia.

## Data Availability Statement

The raw data supporting the conclusions of this article will be made available by the authors, without undue reservation.

## Ethics Statement

This study was approved by the Ethics Committee of Tongji Hospital, Tongji medical college, Huazhong University of Science and Technology (TJ-IRB20190417). Written informed consent to participate in this study was provided by the participants’ legal guardian/next of kin.

## Author Contributions

LL, ML, YX, and PW designed this study and wrote the manuscript. LL, ML, XC, MZ, and YL performed the experiments and collected the data. LL, ML, and XC performed the statistical analysis. All authors contributed to the article and approved the submitted version.

## Conflict of Interest

The authors declare that the research was conducted in the absence of any commercial or financial relationships that could be construed as a potential conflict of interest.

## Publisher’s Note

All claims expressed in this article are solely those of the authors and do not necessarily represent those of their affiliated organizations, or those of the publisher, the editors and the reviewers. Any product that may be evaluated in this article, or claim that may be made by its manufacturer, is not guaranteed or endorsed by the publisher.

## Footnotes

1
http://fiji.sc/

## Supplementary Material

Supplementary Figure 1 Flow chart of image processing.
