# Supplementary figures and images for: Macular Retinal Microvasculature of Hyperopia, Emmetropia, and Myopia in Children
Source: Front Med (Lausanne). 2022 May 20;9:900486. doi: 10.3389/fmed.2022.900486 (PMC9163362; doi:10.3389/fmed.2022.900486)

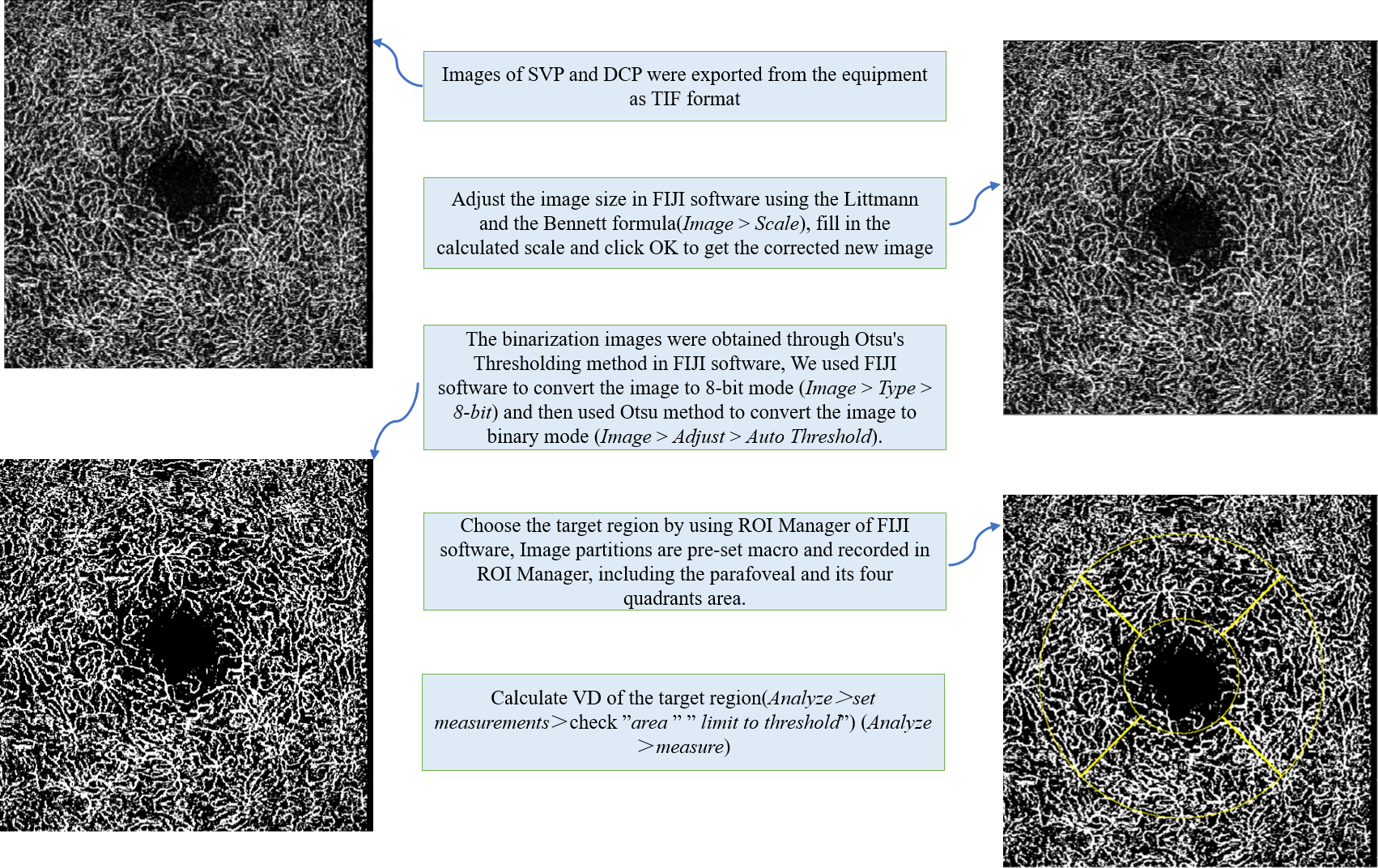

Supplement: Supplementary Figure 1 — Flow chart of image processing. [file Image_1.TIF]
